# Tranexamic acid dose–response relationship for antifibrinolysis in postpartum haemorrhage during Caesarean delivery: TRACES, a double-blind, placebo-controlled, multicentre, dose-ranging biomarker study

**DOI:** 10.1016/j.bja.2022.08.033

**Published:** 2022-10-13

**Authors:** Anne-Sophie Ducloy-Bouthors, Sixtine Gilliot, Maeva Kyheng, David Faraoni, Alexandre Turbelin, Hawa Keita-Meyer, Agnès Rigouzzo, Gabriela Moyanotidou, Benjamin Constant, Francoise Broisin, Agnès L. Gouez, Rémi Favier, Edith Peynaud, Louise Ghesquiere, Gilles Lebuffe, Alain Duhamel, Delphine Allorge, Sophie Susen, Benjamin Hennart, Emmanuelle Jeanpierre, Pascal Odou, the TRACES working group

**Affiliations:** 1 Obstetric Anaesthesia and Intensive Care Unit, Jeanne de Flandre Women's Hospital, Lille University Medical Centre, Lille, France; 2 Groupe de Recherche sur les formes Injectables et les Technologies Associées, ULR 7365, Université de Lille, Lille, France; 3 Département de Biostatistiques, Lille University Medical Centre, Lille, France; 4 METRICS: évaluation des technologies de santé et des pratiques médicales, ULR 2694, Université de Lille, Lille, France; 5 Baylor College of Medicine, Texas Children's Hospital, Houston, TX, USA; 6 Anaesthesia and Intensive Care Unit, Louis Mourier Hospital, Assistance Publique–Hôpitaux de Paris, Colombes, France; 7 Anaesthesia and Intensive Care Unit, Trousseau Hospital, Assistance Publique–Hôpitaux de Paris, Paris, France; 8 Anaesthesia and Intensive Care Unit, Les Bluets Women's Hospital, Assistance Publique–Hôpitaux de Paris, Colombes, France; 9 Anaesthesia and Intensive Care Unit, Seclin General Hospital, Seclin, France; 10 Anaesthesia and Intensive Care Unit, Croix Rousse Lyon Academic Hospital, Lyon, France; 11 Anaesthesia and Intensive Care Unit, Béclère Hospital, Assistance Publique–Hôpitaux de Paris, Clamart, France; 12 Haemostasis Unit, Haematological Laboratory, Armand Trousseau Children's Hospital, Assistance Publique–Hôpitaux de Paris, Paris, France; 13 Haemostasis Unit, Haematological Laboratory, Louis Mourier Hospital, Assistance Publique–Hôpitaux de Paris, Colombes, France; 14 Obstetric Unit, Jeanne de Flandre Women's Hospital, Lille University Medical Centre, Lille, France; 15 Anaesthesia and Intensive Care Unit, Lille University Medical Centre, Lille, France; 16 Toxicology Unit, Biology and Pathology Centre, Lille University Medical Centre, Lille, France; 17 Haemostasis Unit, Biology and Pathology Centre, Lille University Medical Centre, Lille, France

**Keywords:** antifibrinolytic drug, D-dimer, fibrinogen, fibrinolysis, plasmin, plasmin–antiplasmin complex, postpartum haemorrhage, thrombin, tranexamic acid

## Abstract

**Background:**

The optimal dose of tranexamic acid to inhibit hyperfibrinolysis in postpartum haemorrhage is unclear. Tranexamic Acid to Reduce Blood Loss in Hemorrhagic Cesarean Delivery (TRACES) was a double-blind, placebo-controlled, randomised, multicentre dose-ranging study to determine the dose–effect relationship for two regimens of intravenous tranexamic acid *vs* placebo.

**Methods:**

Women experiencing postpartum haemorrhage during Caesarean delivery were randomised to receive placebo (*n*=60), tranexamic acid 0.5 g (*n*=57), or tranexamic acid 1 g i.v. (*n*=58). Biomarkers of fibrinolytic activation were assayed at five time points, with inhibition of hyperfibrinolysis defined as reductions in the increase over baseline in D-dimer and plasmin–antiplasmin levels and in the plasmin peak time.

**Results:**

In the placebo group, hyperfibrinolysis was evidenced by a mean increase over baseline [95% confidence interval] of 93% [68–118] for D-dimer level at 120 min and 56% [25–87] for the plasmin–antiplasmin level at 30 min. A dose of tranexamic acid 1 g was associated with smaller increases over baseline (D-dimers: 38% [13–63] [*P*=0.003 *vs* placebo]; plasmin–antiplasmin: –2% [–32 to 28] [*P*=0.009 *vs* placebo]). A dose of tranexamic acid 0.5 g was less potent, with non-significant reductions (D-dimers: 58% [32–84] [*P*=0.06 *vs* placebo]; plasmin–antiplasmin: 13% [18–43] [*P*=0.051]). Although both tranexamic acid doses reduced the plasmin peak, reduction in plasmin peak time was significant only for the 1 g dose of tranexamic acid.

**Conclusions:**

Fibrinolytic activation was significantly inhibited by a dose of intravenous tranexamic acid 1 g but not 0.5 g. Pharmacokinetic–pharmacodynamic modelling of these data might identify the best pharmacodynamic monitoring criteria and the optimal tranexamic acid dosing regimen for treatment of postpartum haemorrhage.

**Clinical trial registration:**

NCT 02797119.

**Editor's key points**

• Hyperfibrinolysis can occur in postpartum haemorrhage after Caesarean delivery as evident in elevation of D-dimer and plasmin–antiplasmin levels.
• In the TRACES biomarker study, a dose of at least tranexamic acid 1 g i.v. was needed to inhibit activation fibrinolysis.
• Pharmacokinetic–pharmacodynamic modelling of these data and further studies are required to identify the optimal tranexamic acid dosing regimen for treatment of postpartum haemorrhage.

Postpartum haemorrhage (PPH) is the leading cause of maternal death worldwide.^1^ The antifibrinolytic drug tranexamic acid (TXA) reduces bleeding and the need for transfusion during major surgery or trauma.^2^ In the context of PPH after vaginal delivery, a high (4 g) dose of TXA was shown to (i) decrease the volume and duration of blood loss, transfusion needs, and maternal morbidity; and (ii) rapidly inhibit fibrinolysis as quantified by a smaller increase over baseline in plasma D-dimer level.^3^^,^^4^ In the international World Maternal Antifibrinolytic (WOMAN) trial, administration of a single 1 g dose of TXA within 3 h of PPH onset (repeated once if necessary) was associated with a lower haemorrhage-related mortality rate.^5^ TXA appeared to be safe because the incidence of serious adverse events (thromboembolism or seizure) was not elevated.^5^ Prophylactic use of TXA 1 g before vaginal or Caesarean delivery has also been studied.^6^^,^^7^ Ahmadzia and colleagues^8^ recommended a dose of 600 mg to prevent PPH after elective non-haemorrhagic Caesarean delivery. The Tranexamic Acid to Reduce Blood Loss in Hemorrhagic Cesarean Delivery (TRACES) biomarkers study was a double-blind, placebo-controlled, randomised dose-ranging study that includes patients experiencing PPH during Caesarean delivery based on laboratory outcomes.^9^^,^^10^ After the validation of the innovative assays in the TRACES pilot study, we compared two TXA dosing regimens (a standard dose of 1 g and a low dose of 0.5 g) with placebo for inhibition of fibrinolysis, as quantified by changes over time in haemostatic variables.

## Methods

The TRACES trial was approved by a competent national authority (French Drug Safety Agency; reference: 2015–00249926) and an independent ethics committee (CPP North Ouest France; reference 15/50 020216) before initiation of the study, in accordance with Article L1121-4 of the French Public Health Code. This trial was registered on June 13, 2016, at ClinicalTrials.gov (NCT 02797119), in accordance with the French legislation on biomedical research.

The protocol of the TRACES trial has been described in detail elsewhere.^9^^,^^10^ The TRACES pilot study (*n*=22) was conducted in 2014 to validate the simultaneous thrombin–plasmin assay and the TXA dosage (*n*=9), and to create a preliminary pharmacokinetic (PK) model.^10^^,^^11^ The trial was conducted between March 2016 and December 2019 in eight centres in France, all of which complied strictly with French national guidelines on the management of PPH.^12^ An external data and safety monitoring board (DSMB) reviewed the study's scientific quality from the design stage through to the data analysis stage. After publication of the WOMAN trial, the DSMB recommended to focus on biomarkers and the pharmacokinetic–pharmacodynamic (PK/PD) study because the trial was not powered for important clinical outcomes. The funding agencies had no influence on data collection, analysis, interpretation, or reporting.

### Study design

All participants received comprehensive information about the study and gave their written consent before Caesarean delivery. Patients were included in the biomarker study if they experienced a bleeding volume ≥800 ml after Caesarean delivery and had available assay data on at least two D-dimer measurements.^10^ The exclusion criteria, randomisation procedures, and preparation of drug and placebo for administration have been described elsewhere.^10^

### Outcomes

Primary outcome was the difference in the increase over baseline in plasma D-dimer level 120 min after the start of the infusion between treatment and placebo. Secondary outcomes were the treatment *vs* placebo differences in plasma D-dimer (Immunoturbidimetric, STA-LIAtest®, Diagnostica STAGO, Asnières-sur-Seine, France) and plasmin–antiplasmin (PAP) complex level increase over baseline (**TECHNOZYM® PAP Complex ELISA Kit**; Technoclone, Vienna, Austria) at four time points, plasmin generation (PG), thrombin generation (TG),^13^, ^14^, ^15^, ^16^ haemostatic variables (fibrinogen, fibrin monomers, factor II, factor V, antithrombin, and thrombin–antithrombin complexes), and the clinical outcomes at each time point.^10^^,^^17^

### Data management and statistical methods

#### Data management

The study team included the coordinating investigator, the study sponsors' representatives, and the lead investigators from each centre. Informed consent and data collection procedures were monitored by the sponsor. Study data were reviewed in a blind manner by an independent DSMB.

#### Sample size

The sample size calculation of 48 patients per group was based on an expected 30% difference in the treated *vs* placebo groups for the increase over baseline in D-dimer level at T120.^4^^,^^10^

#### Statistical analysis

All statistical analyses were performed by the University of Lille's Biostatistics Department using SAS 9.4 software (SAS Institute Inc., Cary, NC, USA). All tests were two-tailed. The threshold for statistical significance was set to *P*<0.05. Analyses were performed on an intention-to-treat basis for all patients, including those who received a rescue dose of TXA after PPH of >1500 ml. Baseline characteristics were described for each group.

Quantitative variables are expressed as mean (standard deviation [sd]) when normally distributed or median (inter-quartile range [IQR]) otherwise. Categorical variables are expressed as frequency (percentage). Normality of distributions was assessed using histograms and the Shapiro–Wilk test.

Intergroup comparisons of D-dimer and PAP complex levels at each time (30, 60, 120, and 360 min) were performed using a non-parametric analysis of variance. Intergroup comparisons of changes in biochemical parameters between baseline and other times (30, 120, and 360 min) were performed using the constrained longitudinal data analysis (cLDA) model with centre as a random effect.^18^^,^^19^ For clinical variables and variables related to PPH treatments, placebo and treatment groups were compared using the cLDA model with centre as a random effect or (for quantitative longitudinal outcomes) analysis of covariance adjusted for baseline values.^19^, ^20^, ^21^ Binary variables were compared using a mixed logistic regression model, including treatment as fixed effect and centre as random effect (adjusted odds ratios were calculated as a measure of treatment effect size). Linear mixed regression model with centre as a random effect was used to compare groups with regard to non-longitudinal quantitative outcomes. If the model's residuals were not normally distributed (even after logarithmic transformation), non-parametric analysis was applied. Serious and non-serious adverse events were recorded up to Day 42 after delivery for each group.

## Results

**Fig 1:**
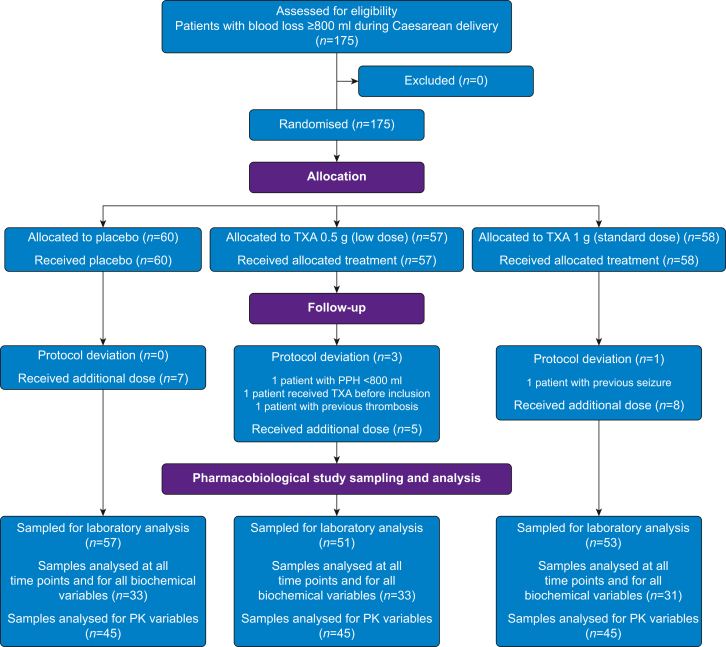
Study flow chart. PK, pharmacokinetic; PPH, postpartum haemorrhage; TXA, tranexamic acid.

There were 151 patients experiencing haemorrhagic Caesarean delivery with assay data on at least two D-dimer measurements included in the TRACES biomarkers trial by the eight centres between 2016 and 2019 (57, 51, and 53 subjects in the placebo, low dose, and standard dose groups, respectively) (Fig. 1). Protocol deviations were noted for four subjects. Baseline subject characteristics and obstetrical data are summarised in Table 1. One or more rescue doses were given to seven (11%) subjects in the placebo group, five (9%) in the low, and eight (14%) in the standard dose groups attributable to PPH >1500 ml, for a total median [IQR] weighted-based dosing of 0 [0–7.4], 6.2 [3.5–17.6], and 12.5 [8–23.3] mg kg^−1^, respectively.

**Table 1:** Baseline anthropometric, obstetric, and bleeding characteristics, by group. CD, Caesarean delivery; IQR, inter-quartile range; PPH, postpartum haemorrhage; TXA, tranexamic acid. Values are quoted as the mean (standard deviation), unless otherwise indicated. T0, at inclusion (baseline); T1, at the end of double-blind TXA or placebo infusion.

	Placebo (*n*=60)	TXA 0.5 g (*n*=58)	TXA 1 g (*n*=57)
Age (yr)	33.1 (5.8) [21–42]	33.7 (5.5) [23–46]	33.2 (4.2) [23–42]
Weight before pregnancy (kg)	73.2 (21.3)	73.8 (21.3)	75.5 (18.8)
Weight at the end of pregnancy (kg)	84.6 (19.6)	85.3 (19.7)	86.3 (15.2)
Height (cm)	163.0 (7.2)	165.1 (7.5)	165.3 (7.0)
BMI (kg m^−2^)	31.6 (6.7)	31.4 (6.5)	31.7 (5.4)
Gestational age (weeks)	38.0 (2.4)	37.8 (2.3)	38.1 (2.2)
Nulliparity, *n* (%)	14 (23)	16 (28)	14 (25)
Illness during pregnancy, *n* (%)	46 (77)	42 (75)	42 (74)
Caesarean delivery indications and causes of postpartum haemorrhage Atony, *n* (%)	45 (75)	41 (71)	41 (72)
Multiple pregnancies, *n* (%)	13 (22)	11 (20)	10 (18)
Previous Caesarean delivery, *n* (%)	19 (32)	14 (24)	15 (26)
Previous postpartum haemorrhage, *n* (%)	9 (15)	12 (21)	8 (15)
Dystocia, *n* (%)	6 (10)	4 (7)	5 (9)
Macrosomia, *n* (%)	7 (12)	4 (7)	3 (5)
Preeclampsia, *n* (%)	1 (2)	2 (3)	2 (3)
Placenta praevia or accreta, *n* (%)	14 (24)	15 (26)	14 (25)
Baseline blood loss at T0 (ml), median (IQR)	902 [852–1100]	1038 [900–1200]	1048 [940–1308]
Delay birth inclusion (min)	39.3 (79.6)	29.9 (62.1)	21.8 (30.5)
Caesarean delivery duration (min), median (IQR)	52 [44–71.5]	63 [48–85]	51.5 [45–69]
General anaesthesia, *n* (%)	2 (3)	3 (5)	3 (5)
Laboratory data			
Haemoglobin (g dl^−1^)	10.6 (1.5)	–10.9 (1.3)	10.6 (1.3)
Fibrinogen (g L^−1^)	4.3 (0.9)	4.5 (0.9)	4.5 (0.8)
D-dimer (ng ml^−1^), median (IQR)	2850 [3130–6780]	2925 [1960–6670]	2950 [2040–3750]
Plasmin–antiplasmin complexes (ng ml^−1^), median (IQR)	441 [302–1161]	452 [312–1224]	403 [295–472]

### Hyperfibrinolysis in the placebo group

Hyperfibrinolysis occurred in the placebo group, identified by an increase over baseline in D-dimer level at 120 min (median increase [IQR]: 4470 [1610–8360] ng ml^−1^), median increase percentage [95% confidence interval {CI}]: 93 [68–118]%), and in PAP level at 30 min (258 [104–507] ng ml^−1^; 56 [25–87]%).

### Inhibition of fibrinolysis by tranexamic acid

**Fig 2:**
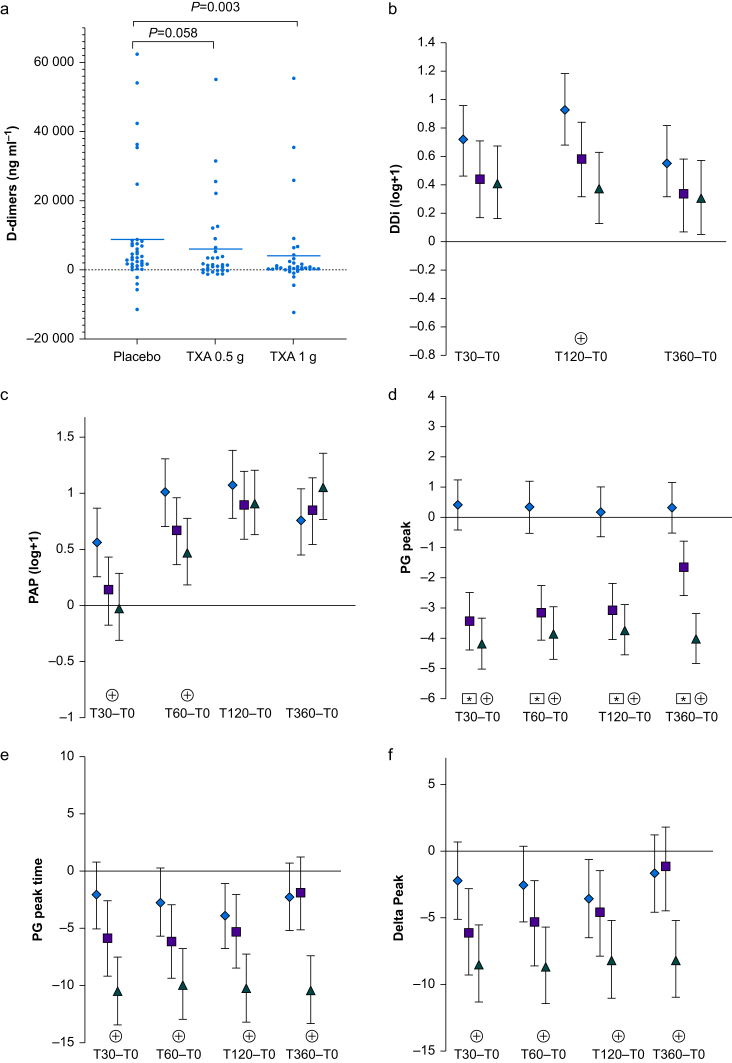
Biomarkers of fibrinolysis activation in the placebo, low dose (TXA 0.5 g), and standard dose (TXA 1 g) groups over baseline. (a) Box plots showing the median [IQR] D-dimer level increase (ng ml^−1^) over baseline at 2 h after infusion in the placebo, low dose (TXA 0.5 g), and standard dose (TXA 1 g) groups. A linear mixed model was used to compare the three groups. (b–f) Laboratory data for the placebo group (black diamonds), the TXA 0.5 g group (dark grey squares), and the TXA 1 g group (light grey triangles). Significant differences between T0 and each time point are indicated by an asterisk (for TXA 0.5 g *vs* placebo) or a star (TXA 1 g *vs* placebo). (b) The D-dimer level increased less from T0 to T120 in the standard dose group than in the placebo group. The figure shows the percentage increase between baseline and T30, T120, and T360 in the placebo (TXA 0 g), low dose (TXA 0.5 g), and standard dose (TXA 1 g) groups. The groups were compared by applying a mixed linear model of covariance. (c) Plasma PAP complex levels increased less from T0 to T30 and T60 in the standard dose group than in the placebo group. The figure shows the percentage increase between baseline and T30, T60, T120, and T360 in the placebo, low dose (TXA 0.5 g), and standard dose (TXA 1 g) groups. The groups were compared by a mixed linear model of covariance. (d) The PG peak from T0 to T30, T60, T120, and T360 was significantly lower in the low dose group (TXA 0.5 g) and the standard dose (TXA 1 g) group than in the placebo group. The groups were compared by a mixed linear model of covariance. (e) The PG peak time decreased more in the standard (TXA 1 g) group than in the placebo group. The figure shows the percentage decrease between baseline and T30, T120, and T360 in the placebo, low dose (TXA 0.5 g), and standard dose (TXA 1 g) groups. The groups were compared by a mixed linear model of covariance. (f) The time interval between the thrombin generation peak and the PG peak decreased significantly more in the standard dose (TXA 1 g) group than in the placebo group. The figure shows the percentage decrease from baseline (T0) to each time point (T30, T60, T120, and T360). The groups were compared by a mixed linear model of covariance. PAP, plasmin–antiplasmin; PG, plasmin generation; TXA, tranexamic acid.

We observed a significant difference between the standard dose TXA group and placebo group with regard to median [IQR] plasma D-dimer level at 120 min (4340 [3240–5960] ng ml^−1^) *vs* 8930 [95% CI: 3820–17 940] ng ml^−1^, respectively; *P*=0.03) (Fig. 2a; Supplementary Table S1). The D-dimer increase over baseline at T120 was lower in the standard dose group (630 [95% CI: 240–2000] ng ml^−1^, with a moderate size effect: Cohen's d=0.51) but not in the low dose group (Supplementary Table S2). The percentage increase over baseline in D-dimer level at T120 was lower in the standard dose group (38% [95% CI: 13–63%] *vs* 93% [95% CI: 68–118%] in the placebo group; *P*=0.003 *vs* placebo) but not in the low dose group (58% [95% CI: 32–84%]; *P*=0.058 *vs* placebo) (Fig. 2b; Supplementary Table S3).

The median [IQR] PAP complex level was lower in the standard dose group than in the placebo group after 30 min (347 [261–485] ng ml^−1^ *vs* 639 [455–199] ng ml^−1^, respectively) and after 60 min (499 [379–1110] ng ml^−1^ *vs* 1160 [717–2530] ng ml^−1^, respectively) (Supplementary Table S1). The percentage increase in PAP from baseline at 30 min in the placebo group (56% [95% CI: 25–87%]) was lower in the standard dose group (–2% [95% CI: –32%–28%]; *P*=0.009) but not in the low dose group (13% [95% CI: –18%–43%]; *P*=0.051) (Fig. 2c; Supplementary Table S3).

The PG peak was lower in the two TXA groups relative to the placebo group at each time point (Fig. 2d; Supplementary Table S4). The time to plasmin peak and the time interval between the TG and PG peaks were lower at each time point (relative to placebo) in the standard dose group but not in the low dose group (Fig 2e and f; Supplementary Table S4). There was no significant impact of the two TXA dose regimens (relative to placebo) on plasma levels of fibrinogen, fibrin monomers, factor II, factor V, antithrombin, and thrombin–antithrombin complexes or the TG potential (as quantified by the TG peak and the area under the curve) (Supplementary Tables S1 and S5).

The median plasma and urine TXA concentrations for the 90 treated subjects are summarised in Table 2. At T360, TXA was totally excreted in both groups.

**Table 2:** Plasma and urine tranexamic acid concentrations. IQR, inter-quartile range.

	Tranexamic acid 0.5 g (*n*=45)	Tranexamic acid 1 g (*n*=45)
Plasma concentration, median [IQR] (mg L^−1^)		
T1	32 [10–46]	75 [55–116]
T30	21 [17–23]	41 [33–48]
T60	15 [13–17]	30 [23–36]
T120	10 [7–11]	18 [15–22]
T360	2 [1–6]	4 [3–6]
Urine concentration, median [IQR] (mg L^−1^)		
T0–T120	1573 [1174–3550]	3198 [2153–3910]
T120–T360	2598 [2020–3435]	4640 [3125–6471]

### Clinical outcomes

Median additional blood loss at 6 h was lower in the standard dose group (134 [95% CI: 50–419] ml) than in the low dose group (300 [95% CI: 68–630] ml; *P*=0.042). However, median additional blood loss in the placebo group (208 [95% CI: 55–539] ml) did not differ from that observed in the standard dose group (*P*=0.35) or in the low dose group (*P*=0.28). There were no differences between the placebo and treated groups with regard to total blood loss at 6 h, duration of bleeding, proportion of subjects with life-threatening blood loss (>2500 ml), fall in haemoglobin level, anaemia, need for transfusion or procoagulant treatment, incidence of invasive procedures, ICU admission rate, or incidence of organ failure (Supplementary Table SC).

### Safety endpoints

Relative to the placebo group, non-serious adverse events (e.g. nausea and vomiting) were more frequent in the standard dose group but not in the low dose group (Supplementary Table SC). There were no seizures, no thrombotic events, no cases of renal failure, and two cases of transient blood creatinine elevation in the placebo group.

## Discussion

The results of the TRACES biomarker study suggest that (i) there was activation of fibrinolysis in PPH after Caesarean delivery, (ii) a dose of TXA 1 g was associated with lower levels (relative to placebo) of hyperfibrinolysis biomarkers, and (iii) a low (0.5 g) dose of TXA was less effective than a standard 1 g dose. Hence, in a context of haemorrhage and intense fibrinolytic activation, one can assume that a dose of at least TXA 1 g must be administered.

### Activation of fibrinolysis and inhibition by tranexamic acid

The mechanisms underlying fibrinolysis activation in PPH have not been extensively characterised. The physiological activation of fibrinolysis is observed 30 min after delivery as evidenced by elevation in plasma D-dimer and PAP levels.^4^, ^5^, ^6^, ^7^, ^8^, ^9^, ^10^, ^11^, ^12^, ^13^, ^14^, ^15^, ^16^, ^17^, ^18^, ^19^, ^20^, ^21^, ^22^ In the context of PPH, there were early but variable increases in plasma D-dimer. In a sub-study of the WOMAN trial, Roberts and colleagues^23^ detected coagulopathy in 38 (23%) out of 167 patients. The mean (sd) D-dimer concentration was 7.1 (7.0) mg L^−1^ in the TXA group (*n*=83) and 9.6 (8.6) mg L^−1^ in the placebo group (*n*=84) (*P*=0.09).^24^

As Miszta and colleagues^16^ demonstrated for three different doses (5, 10, and 15 mg kg^−1^) in 30 elective Caesarean deliveries, the TRACES results suggest that PG peak was lower in both treated groups, although the 1 g dose of TXA (but not 0.5 g) reduced the PG peak time and the time interval between TG and PG peaks.

The results of the TRACES trial suggest that a 1 g dose of TXA was able to inhibit fibrinolytic activation. Indeed, elevated plasma levels of fibrinolysis activation markers tended to be reduced by the standard dose of TXA but not by the low dose. PD modelling will be performed to include the additional doses and their correlation with intensity of fibrinolysis activation and residual TXA plasma concentrations.

### How can the optimal dose of tranexamic acid be determined?

A trial by Li and colleagues^25^ evaluated the efficacy of TXA by targeting a plasma concentration of 10 mg L^−1^ and a reduction in maximal lysis from 100% (after addition of tissue plasminogen activator) to 17% (in the treated plasma). The trial had several limitations. First, the *ex vivo* definition of the treated plasma antifibrinolytic impact did not take into account the high variability of fibrinolytic activation in PPH. Second, the target plasma TXA concentration of 10 mg L^−1^ was defined in an *in vitro* model. Third, the model was not improved by including body weight as a covariable. Fourth, the 15 mg kg^−1^ dose was limited to a maximum total dose of 1 g. And fifth, urine samples were not collected, restricting validation of excreted concentrations.^8^^,^^25^

A PK model has been created from the TRACES data in the context of ongoing haemorrhage. The model was initially based on 53 data points from nine haemorrhagic patients in the TRACES pilot study and then consolidated by analysis of 385 blood samples and 117 urine samples from 79 patients.^10^^,^^11^^,^^26^ A two-compartment model with dual (urinary and non-urinary) first-order excretion of TXA best predicted its plasma and urine concentrations.^11^, ^12^, ^13^, ^14^, ^15^, ^16^, ^17^, ^18^, ^19^, ^20^, ^21^, ^22^, ^23^, ^24^, ^25^, ^26^ Parametric non-linear mixed-effect modelling (Monolix 2020R1, Lixoft, Anthony, France) was computed. Central compartment volume increased with body weight measured before pregnancy. The final model was internally validated using a 550-run bootstrap. The non-urinary excretion pathway can be interpreted as TXA loss into haemorrhaged blood, as hypothesised in cases of massive trauma-induced haemorrhage.^27^ This study supports giving a dose of TXA 1 g and repeating administration in the event of massive haemorrhage and intense fibrinolytic activation as in PPH.

### Is there a dose–response relationship for blood loss reduction?

We did not observe a significant difference in additional blood loss between either of the TXA groups and the placebo group. However, there was a non-significant trend towards lower additional blood loss in the TXA 1 g group relative to the TXA 0.5 g group. Furthermore, there were no significant differences between either of the TXA groups and the placebo group with regard to total blood loss, anaemia, transfusion requirement, maternal morbidity, or well-being. Our results must be interpreted in light of the study sample size, which was smaller than that of the pragmatic WOMAN trial, in which 10 051 patients were randomised to receive TXA 1 g and 10 009 placebo.^5^ In the WOMAN trial, 28% of patients received TXA 2 g, and bleeding-related maternal death rate was significantly lower only when TXA was administered within 3 h of delivery.^5^ In our previous A multi-centre open-label randomised controlled trial measuring the efficacy of tranexamic acid to reduce postpartum haemorrhage volume (EXADELI) study, a high dose of TXA 4 g was clinically effective when given at the onset of PPH.^3^ Similarly, in trauma patients with haemodynamic decompensation, an early (<1 h) repeated dose of TXA 3 g reduced 30 day mortality compared with placebo, whereas a pre-hospital dose of TXA 1 or 2 g did not.^28^ However, administration of TXA 4 g was questioned because of the reported risk of seizure after cardiac surgery, venous thrombotic events in gastrointestinal bleeding, and suspected kidney injury in patients with preeclampsia.^29^, ^30^, ^31^

### Optimal time interval for tranexamic acid administration

The WOMAN trial showed that TXA administration was ineffective after >3 h of PPH.^5^ The HALT-IT Trial Collaborators^30^ explained the presence of an optimal time interval by (i) a lack of effect of TXA on late plasminogen activation by urokinase–plasminogen activator and (ii) the competitive mechanism of action of TXA on alpha-2 plasmin inhibitor. This competitive mechanism might explain the lack of effectiveness of low-dose TXA in the present study. Accordingly, international guidelines recommend TXA administration within 3 h of bleeding onset.^5^^,^^32^

### Impact of tranexamic acid on thrombin generation and plasma fibrinogen levels

The TRACES biomarker study suggests that TXA had no impact on the TG potential or plasma levels of fibrinogen, fibrin monomers, thrombin–antithrombin complexes, or factor II. These findings are consistent with the TG data of Ahmadzia and colleagues.^8^ The EXADELI trial suggested that a high TXA dose was not able to avoid a decrease in plasma fibrinogen levels or to restore them.^3^ The biomarker data from the TRACES trial confirmed that there was no impact of a low or standard dose of TXA on fibrinogen plasma levels; this implies that fibrinogen levels should be restored by administration of plasma, fibrinogen concentrate, or cryoprecipitate concomitantly with fibrinolysis inhibition.

### Study strengths

The TRACES trial had a robust double-blind, placebo-controlled, randomised design. The rigorous, repeated collection of samples provided valuable information and enabled adapting the TXA dose and dosing schedule as a function of hyperfibrinolysis intensity and duration.

### Study limitations

Despite its prospective, randomised design, this study had a number of limitations. First, the sample size (175 patients) might have been too small to demonstrate the clinical efficacy of TXA. Publication of the results of the WOMAN trial prompted us to reconsider the number of participants required, and the DSMB advised us to focus on the biomarker aspect of the protocol. Accordingly, we recruited the more than 144 patients needed to perform the laboratory and PK analyses.

### Conclusions

The TRACES biomarker study shows that a dose of at least tranexamic acid 1 g i.v. was needed to inhibit postpartum haemorrhage-induced hyperfibrinolysis. Pharmacodynamic modelling or further dose-ranging studies should help determine the optimal dose and dosing schedule for use of tranexamic acid in the setting of postpartum haemorrhage after Caesarean delivery.

## Authors' contributions

Study conception/design: ASDB, AD, SS, EJ, BH, EJ

Preliminary studies for specific assays: ASDB, BH, EJ

Preliminary studies of the dose-ranging model: ASDB, SG, BH, PO

Preliminary studies of a simultaneous thrombin–plasmin generation assay: ASDB, SS, EJ

Pharmacokinetic/pharmacodynamic analyses: SG, PO

Elaboration of tranexamic acid assay method: BH

Validation of production of the study drug: BH

Data acquisition: ASDB, AT, HKM, AR, GM, BC, FB, ALG, RF, EP, LG, AD, SS, EJ, BH

Data management: ASDB, AT, HKM, AR, GM, BC, FB, AL-G, RF, EP, LG, AD, SS, EJ, BH

Data analysis: ASDB, SG, MK, GL, AD, SS, EJ, BH, PO

Data interpretation: ASDB, SG, GL, AD, SS, EJ, BH, PO

Article drafting: ASDB, SG, DF, AD, SS, EJ, BH, PO

Article revision: SG, MK, DF, AT, HK-M, AR, GM, BC, FB, AL-G, RF, EP, LG, GL, AD, SS, EJ, BH, PO

Final article revision: ASDB

Final article approval: all authors
