# Supplementary material for: Tranexamic acid dose–response relationship for antifibrinolysis in postpartum haemorrhage during Caesarean delivery: TRACES, a double-blind, placebo-controlled, multicentre, dose-ranging biomarker study
Source: Br J Anaesth. 2022 Oct 13;129(6):937–45. doi: 10.1016/j.bja.2022.08.033 (PMC9748994; doi:10.1016/j.bja.2022.08.033)
Supplement: Multimedia component 1 [file mmc1.docx]

**Appendix  : Results**

- Tables of comparative analysis between the 2 treated groups (TXA 0.5 g low-dose and TXA 1 g standard dose) versus placebo of clinical (Table SC) and biomarkers (Table S1) data and their evolution over baseline (T0 before injection) and each time-point (T30, T60, T120, T360 = 30, 60, 120, 360 minutes after injection) by a linear mixed model of covariance measuring the strengh of the treatment impact on these evolutions; the linear covariance model is significant if the interquartile of the difference does not cross the zero for the quantitative variables and does not cross the 1 for the qualitative variables ; The force of the impact is then measured by the d-Cohen test defined as low from 0,20 to 0,40 ; mils from 0,40 to 0,60 and strong if more than 0,60.
- Description of D-dimers (ng.mL^-1^) and PAP (ng.mL^-1^) levels increase over baseline between T0 and each time-point and comparison of the treated groups versus placebo (Table S2).
- Description of D-dimers and PAP levels increase expressed as percentage over baseline at each time point. D-dimers and PAP reduction by the 2 treatment dose-regimens are expressed as percentage of reduction compared to placebo increase, between T0 and T120 and between T0 and the other time-points respectively (Table S3).
- Description of the plasmin generation increase (peak) and time to peak decrease between T0 and each time-point and comparison of the treated groups versus placebo (Table S4).
- Description of the thrombin generation increase (area under the curve) and time to peak decrease between T0 and each time-point and comparison of the treated groups versus placebo (Table S5).

**Table S1 : Biomarkers data at each time points and their changes over time in the TXA 0.5 g group vs placebo and in the TXA 1 g group vs placebo.**

|  |  |  |  |  |  |  |  |  |  |  |  |
| --- | --- | --- | --- | --- | --- | --- | --- | --- | --- | --- | --- |
|  |  | Groups | |  |  |  |  |  | Effect size (Difference from T0) | |  |
|  |  |  |  |  |  |  |  | **Mean difference**  **(CI95%CI)** | **Cohen’s d** | **Mean difference**  **(CI95%CI)** | **Cohen’s d** |
| Parameters | **Time** | **n** | **Placebo** | **n** | **TXA 0.5** | **n** | **TXA 1** | **TXA 0.5 vs Placebo** |  | **TXA 1 vs placebo** |  |
| Biological criteria |  |  |  |  |  |  |  |  |  |  |  |
| D-dimers (ng.mL^-1^) mean (SD) | T30 | 35 | 6130  (2910 to 13360) | 31 | 4270  (2860 to 15200) | 33 | 3600  (2880 to 7300) | -5.64 (-22.10 to 10.81)^a^ | 0.13 | -12.50 (-28.69 to 3.69) ^a^ | 0.42 |
|  | T120 | 34 | 8930  (3820 to 17940) | 34 | 4550  (3180 to 15180) | 37 | 4340  (3240 to 5960) | -15.67 (-32.47 to 1.35) ^a^ | 0.24 | -29.87 (-46.54 to -13.21) ^a^ | 0.51 |
|  | T360 | 37 | 6130  (4001 to 11280) | 36 | 4240  (2795 to 99985) | 35 | 3290  (2290 to 5410) | -12.70 (-29.73 to 4.32) ^a^ | 0.35 | -24.65 (-41.79 to -7.50) ^a^ | 0.68 |
|  |  |  |  |  |  |  |  |  |  |  |  |
| PAP complexes (ng.mL^-1^) mean (SD) | T30 | 34 | 639  (455 to 1991) | 34 | 496  (349 to 1273) | 34 | 347  (261 to 485) | -13.53 (-32.83 to 5.77) ^a^ | 0.15 | -40.16 (-59.46 to -20.86) ^a^ | 0.61 |
|  | T60 | 35 | 1158  (717 to 2532) | 35 | 836  (505 to 1629) | 34 | 499  (379 to 1107) | -12.90 (-31.85 to 6.05) ^a^ | 0.06 | -33.03 (-52.11 to -13.94) ^a^ | 0.40 |
|  | T120 | 37 | 1238  (695 to 1885) | 36 | 922  (615 to 2531) | 38 | 603  (480 to 1680) | -5.22 (-25.34 to 14.91) ^a^ | 0.12 | -25.04 (-44.90 to -5.19) ^a^ | 0.30 |
|  | T360 | 38 | 1033  (572 to 2020) | 38 | 880  (622 to 2261) | 37 | 760  (530 to 1852) | 5.42 (-15.01 to 25.85) ^a^ | 0.08 | -4.21 (-24.78 to 16.36) ^a^ | 0.04 |
|  |  |  |  |  |  |  |  |  |  |  |  |
| Haemoglobin, (g.dL^-1^) mean (SD) | T360 | 44 | 10.1 (1.2) | 39 | 10.2 (1.4) | 39 | 10.3 (1.4) |  |  |  |  |
|  | T30-T0 | 37 | -0.58 (1.22) | 33 | -0.97 (0.94) | 33 | -0.86 (0.89) | -0.34 (-0.82 to 0.15) ^b^ | 0.35 | -0.25 (-0.73 to 0.24) ^b^ | 0.25 |
|  | T120-T0 | 37 | -0.22 (1.61) | 34 | -0.64 (1.11) | 37 | -0.35 (0.99) | -0.22 (-0.73 to 0.29) ^b^ | 0.20 | -0.13 (-0.64 to 0.37) ^b^ | 0.08 |
|  | T360-T0 | 39 | -0.51 (1.00) | 37 | -0.65 (1.13) | 37 | -0.40 (1.34) | -0.06 (-0.51 to 0.39) ^b^ | 0.04 | 0.16 (-0.29 to 0.61) ^b^ | 0.14 |
|  |  |  |  |  |  |  |  |  |  |  |  |
| Plaquettes (10^9^.L^-1^) mean (SD) | T360 | 44 | 185 (54.1) | 39 | 200 (66.3) | 39 | 222 (63.3) |  |  |  |  |
|  | T360-T0 | 39 | -1.2 (31.0) | 37 | -4.6 (29.7) | 37 | -6.4 (44.6) | -1.76 (-17.06 to 13.54) ^b^ | 0.03 | 1.11 (-14.11 to 16.33) ^b^ | 0.08 |
|  |  |  |  |  |  |  |  |  |  |  |  |
|  |  |  |  |  |  |  |  |  |  |  |  |
| Creatininemia (mg.L^-1^) mean (SD) | T360 | 42 | 6.3 (2.5) | 38 | 6.0 (1.7) | 37 | 5.9 (1.6) |  |  |  |  |
|  |  |  |  |  |  |  |  |  |  |  |  |
| Fibrinogen (g.L^-1^) mean (SD) | T360 | 43 | 3.9 (1.0) | 39 | 4.0 (0.9) | 39 | 4.1 (0.9) |  |  |  |  |
|  | T30-T0 | 39 | -0.33 (0.57) | 33 | -0.58 (0.62) | 33 | -0.64 (0.65) | -0.19 (-0.48 to 0.09) ^b^ | 0.35 | -0.20 (-0.48 to 0.08) ^b^ | 0.49 |
|  | T120-T0 | 39 | -0.42 (0.69) | 35 | -0.55 (0.63) | 36 | -0.37 (0.71) | -0.03 (-0.33 to 0.27) ^b^ | 0.14 | 0.14 (-0.16 to 0.43) ^b^ | 0.12 |
|  | T360-T0 | 40 | -0.23 (0.57) | 36 | -0.52 (0.66) | 38 | -0.27 (0.78) | -0.20 (-0.49 to 0.09) ^b^ | 0.40 | 0.05 (-0.23 to 0.34) ^b^ | 0.01 |
|  |  |  |  |  |  |  |  |  |  |  |  |
| FII (I./ml^-1^) mean (SD) | T360 | 41 | 97.2 (13.1) | 38 | 100 (14.6) | 38 | 101 (18.4) |  |  |  |  |
| FV (IU.m^-1^l) mean (SD) | T360 | 41 | 97.4 (23.5) | 38 | 100 (24.3) | 38 | 96.0 (27.3) |  |  |  |  |
| Antithrombin (IU.ml^-1^) mean (SD) | T360 | 39 | 83.7 (12.2) | 38 | 86.6 (14.5) | 36 | 86.0 (12.9) |  |  |  |  |
| Fibrin monomer (µg.L^-1^) mean (SD) | T360 | 38 | 112  (33.0 to 151) | 38 | 88.5  (32.0 to 151) | 37 | 55.0  (24.0 to 134) |  |  |  |  |
| TAT (µg.L^-1^) mean (SD) | T360 | 38 | 17.8  (9.8 to 30.6) | 37 | 12.3  (9.3 to 29.3) | 37 | 12.1  (6.1 to 17.2) |  |  |  |  |
|  |  |  |  |  |  |  |  |  |  |  |  |

Values are expressed in median (IQR) unless otherwise indicated. ^a^Estimate (95%CI) obtained using non-parametric ANCOVA, ^b^Estimates (95%CI) obtained using cLDA analysis with center as random effect.

**Table S2 : D-dimers and PAP levels increase over baseline at each time-point and comparison of the TXA treated groups versus placebo**

| Parameters | Time | Placebo | TXA 0.5 | TXA 1 | TXA 0.5 vs Placebo  Mean difference (SE) | Cohen’s d | TXA 1 vs Placebo  Mean difference (SE) | Cohen’s d |
| --- | --- | --- | --- | --- | --- | --- | --- | --- |
| D-dimers | T30-T0 | 1750  (600 to 8900) | 870  (-50.0 to 6630) | 680  (80 to 2150) | -16.03 (-31.23 to -0.84) | 0.51 | -17.57 (-32.74 to -2.39) | 0.59 |
| (ng.mL^-1^) | T120-T0 | 4470  (1610 to 8360) | 1260  (-150 to 5915) | 630  (240 to 2000) | -21.49 (-36.39 to -6.59) | 0.69 | -28.85 (-43.42 to -14.28) | 1.04 |
|  | T360-T0 | 2060  (260 to 5690) | 780  (-1010 to 2041) | 220  (-550 to 1480) | -18.11 (-32.92 to -3.30) | 0.28 | -20.39 (-35.44 to -5.34) | 0.66 |
|  |  |  |  |  |  |  |  |  |
| PAP | T30-T0 | 258  (104 to 507) | 0  (-57.0 to 124) | -47.0  (-103 to 53.0) | -40.03  (-58.76 to -21.29) | 1.06 | -48.85  (-67.75 to -29.95) | 1.27 |
| (ng.mL^-1^) | T60-T0 | 537  (208 to 1486) | 171  (-2.0 to 684) | 89.0  (-23.0 to 281) | -27.29  (-46.61 to -7.97) | 0.70 | -36.74  (-56.15 to -17.33) | 0.94 |
|  | T120-T0 | 667  (284 to 119) | 324  (81.5 to 753) | 241  (84.5 to 1105) | -18.67  (-32.27 to 1.94) | 0.43 | -21.82  (-42.28 to -1.16) | 0.51 |
|  | T360-T0 | 285  6.0 to 822) | 386  (124 to 1485) | 399  (137 to 1542) | 7.20  (-12.99 to 27.39) | 0.17 | 11.74  (-8.87 to 32.37) | 0.27 |

Measured values of the increase over baseline are expressed in median (IQR). Estimates (95%CI) obtained using. Estimate (95%CI) obtained using non-parametric ANCOVA adjusted on T0 values.

**Table S3 : D-dimer level increase at T120 and PAP level increase at each time points over baseline in each groups expressed as percentage and their changes in the treated groups vs placebo.**

| Parameters | |  | | Placebo | | TXA 0.5 | | TXA 1 | TXA 0.5 vs Placebo  Mean difference (SE) | | p | | TXA 1 vs Placebo  Mean difference (SE) | | p | |  |
| --- | --- | --- | --- | --- | --- | --- | --- | --- | --- | --- | --- | --- | --- | --- | --- | --- | --- |
| D-dimers | | T0-T120 | | 0.93  (0.68 to 1.18) | | 0.58  (0.32 to 0.84) | | 0.38  (0.13 to 0.63) | -0.35  (-0.71 to 0.01) | | 0.058 | | -0.55  (-0.9 to -0.19) | | 0.003 | |  |
| PAP | T0-T30 | | 0.56  (0.25 to 0.87) | | 0.13  (-0.18 to 0.43) | | -0.02  (-0.32 to 0.28) | | | -0.43  (-0.87 to 0) | | 0.051 | | -0.58  (-1.01 to -0.15) | | 0.009 | |
|  | T0-T60 | | 1.01  (0.7 to 1.31) | | 0.66  (0.36 to 0.96) | | 0.48  (0.18 to 0.78) | | | -0.35  (-0.77 to 0.08) | | 0.11 | | -0.52  (-0.96 to -0.09) | | 0.017 | |
|  | T0-T120 | | 1.08  (0.78 to 1.38) | | 0.89  (0.59 to 1.19) | | 0.91  (0.62 to 1.2) | | | -0.19  (-0.61 to 0.23) | | 0.37 | | -0.17  (-0.58 to 0.25) | | 0.43 | |
|  | T0-T360 | | 0.75  (0.45 to 1.04) | | 0.84  (0.54 to 1.13) | | 1.06  (0.77 to 1.36) | | | 0.09  (-0.33 to 0.51) | | 0.67 | | 0.32  (-0.1 to 0.74) | | 0.14 | |

Values are expressed in median (IQR). Estimate (95%CI) obtained using non-parametric ANCOVA adjusted on T0 values.

**Table S4 : Plasmin generation (PG) peak increase and time to peak decrease at each time points over baseline in each groups expressed as percentage and their changes in the treated groups vs placebo.**

| Parameters | Time | Placebo | TXA 0.5 | TXA 1 | TXA 0.5 vs Placebo  Mean difference (SE) | p | TXA 1 vs Placebo  Mean difference (SE) | p |
| --- | --- | --- | --- | --- | --- | --- | --- | --- |
| PG peak | T0-T30 | 0.4  (-0.44 to 1.23) | -3.45  (-4.39 to -2.51) | -4.18  (-5.04 to -3.33) | -3.85  (-5.1 to -2.59) | <.001 | -4.58  (-5.77 to -3.38) | <.001 |
| (nM) | T0-T60 | 0.33  (-0.53 to 1.19) | -3.17  (-4.08 to -2.25) | -3.85  (-4.73 to -2.97) | -3.5  (-4.75 to -2.24) | <.001 | -4.18  (-5.41 to -2.95) | <.001 |
|  | T0-T120 | 0.17  (-0.66 to 1) | -3.12  (-4.04 to -2.19) | -3.73  (-4.57 to -2.89) | -3.29  (-4.53 to -2.04) | <.001 | -3.9  (-5.09 to -2.72) | <.001 |
|  | T0-T360 | 0.3  (-0.54 to 1.15) | -1.68  (-2.58 to -0.78) | -4.01  (-4.86 to -3.16) | -1.98  (-3.22 to -0.75) | 0.002 | -4.31  (-5.51 to -3.12) | <.001 |
|  |  |  |  |  |  |  |  |  |
| PG peak time | T0-T30 | -2.16  (-5.1 to 0.77) | -5.93  (-9.21 to -2.65) | -10.53  (-13.49 to -7.56) | -3.77  (-8.17 to 0.63) | 0.093 | -8.36  (-12.54 to -4.19) | <.001 |
| (min) | T0-T60 | -2.7  (-5.71 to 0.31) | -6.17  (-9.37 to -2.96) | -9.91  (-13 to -6.82) | -3.47  (-7.87 to 0.93) | 0.12 | -7.21  (-11.53 to -2.9) | 0.001 |
|  | T0-T120 | -3.92  (-6.84 to -1) | -5.28  (-8.53 to -2.04) | -10.27  (-13.21 to -7.32) | -1.36  (-5.73 to 3) | 0.54 | -6.34  (-10.49 to -2.2) | 0.003 |
|  | T0-T360 | -2.27  (-5.21 to 0.68) | -1.96  (-5.14 to 1.21) | -10.43  (-13.4 to -7.45) | 0.3  (-4.03 to 4.63) | 0.89 | -8.16  (-12.34 to -3.97) | 0.001 |
|  |  |  |  |  |  |  |  |  |
| Time interval between TG and PG peaks | T0-T30 | -2.3  (-5.17 to 0.57) | -6.12  (-9.31 to -2.93) | -8.46  (-11.33 to -5.59) | -3.82  (-8.11 to 0.47) | 0.081 | -6.16  (-10.22 to -2.1) | 0.003 |
| (min) | T0-T60 | -2.59  (-5.46 to 0.28) | -5.47  (-8.66 to -2.28) | -8.63  (-11.5 to -5.76) | -2.88  (-7.17 to 1.41) | 0.19 | -6.04  (-10.1 to -1.97) | 0.004 |
|  | T0-T120 | -3.67  (-6.55 to -0.8) | -4.74  (-7.93 to -1.55) | -8.18  (-11.05 to -5.31) | -1.07  (-5.36 to 3.22) | 0.62 | -4.51  (-8.57 to -0.45) | 0.030 |
|  | T0-T360 | -1.76  (-4.64 to 1.11) | -1.35  (-4.54 to 1.84) | -8.16  (-11.03 to -5.29) | 0.42  (-3.88 to 4.71) | 0.85 | -6.4  (-10.46 to -2.34) | 0.002 |

Values are expressed in median (IQR). Estimate (95%CI) obtained using non-parametric ANCOVA adjusted on T0 values.

**Table S5 : Thrombin generation (TG) area under the curve increase and time to peak decrease percentage over T0 at each time-point and comparison of the treated groups versus placebo**

| Parameters | Time | Placebo | TXA 0.5 | TXA 1 | TXA 0.5 vs Placebo  Mean difference (SE) | p | TXA 1 vs Placebo  Mean difference (SE) | p |
| --- | --- | --- | --- | --- | --- | --- | --- | --- |
| TG AUC  (nM.min) | T0-T30 | 1295  (-3651 to 6241) | 1802  (-681 to 4285) | 2314  (-321 to 4950) | 507  (-5027 to 6042) | 0.86 | 1019  (-4585 to 6624) | 0.72 |
|  | T0-T60 | 808  (-721 to 2339) | 232  (-1442 to 1906) | 938  (-663 to 2541) | -576.93  (-2845.63 to 1691.76) | 0.62 | 129.67  (-2086.38 to 2345.71) | 0.91 |
|  | T0-T120 | 1284.25  (-305.42 to 2873.91) | 300.77  (-1375.97 to 1977.52) | 1108.09  (-487.19 to 2703.37) | -983.47  (-3293.99 to 1327.05) | 0.40 | -176.16  (-2428.26 to 2075.94) | 0.88 |
|  | T0-T360 | 895.66  (-613.37 to 2404.69) | -257.58  (-1933.36 to 1418.19) | 180.72  (-1359.95 to 1721.4) | -1153.25  (-3408.33 to 1101.84) | 0.32 | -714.94  (-2871.53 to 1441.64) | 0.51 |
| TG peak time  (min) | T0-T30 | -0.01  (-0.66 to 0.65) | -0.2  (-0.93 to 0.52) | -0.46  (-1.15 to 0.24) | -0.2  (-1.17 to 0.78) | 0.69 | -0.45  (-1.41 to 0.51) | 0.35 |
|  | T0-T60 | -0.45  (-1.12 to 0.23) | -0.42  (-1.14 to 0.31) | -1.16  (-1.86 to -0.46) | 0.03  (-0.96 to 1.02) | 0.95 | -0.71  (-1.68 to 0.26) | 0.15 |
|  | T0-T120 | -0.6  (-1.25 to 0.05) | -0.6  (-1.32 to 0.13) | -1.15  (-1.82 to -0.48) | 0  (-0.97 to 0.97) | 0.99 | -0.55  (-1.48 to 0.38) | 0.24 |
|  | T0-T360 | -0.93  (-1.59 to -0.27) | -1.45  (-2.15 to -0.75) | -1.27  (-1.95 to -0.59) | -0.52  (-1.48 to 0.45) | 0.29 | -0.34  (-1.29 to 0.6) | 0.48 |

Values are expressed in median (IQR). Estimate (95%CI) obtained using non-parametric ANCOVA adjusted on T0 values.

**Table SC : Evolution of the clinical parameters and PPH treatments by groups.**

|  |  | Groups | | | | | | Effect size (Difference from T0) | | | | |
| --- | --- | --- | --- | --- | --- | --- | --- | --- | --- | --- | --- | --- |
|  |  |  |  |  |  |  |  | **Mean difference**  **(CI95%CI)** | **Cohen’s d** | **Mean difference**  **(CI95%CI)** | | **Cohen’s d** |
| Parameters | **Time** | **n** | **Placebo** | **n** | **TXA 0.5** | **n** | **TXA 1** | **TXA 0.5 vs Placebo** | | **TXA 1 vs placebo** | | |
| Clinical |  |  |  |  |  |  |  |  | |  | | |
| Blood loss volume |  |  |  |  |  |  |  |  |  |  |  | |
| Additional (mL) | T360-T0 | 60 | 208  [55.0 to 539] | 57 | 300  [68.0 to 630] | 58 | 134  [50.0 to 419] | 12.12  [-13.22 to 37.46] ^a^ | 0.15 | -14.15  [-39.75 to 11.44] ^a^ | 0.12 | |
|  |  |  |  |  |  |  |  |  |  |  |  | |
| Total (mL) | T360 | 60 | 1213  [985 to 1577] | 57 | 1400  [1070 to 1820] | 58 | 1317  [1080 to 1570] | 12.71  [-6.13 to 31.54]^b^ | 0.24 | 8.84  [-9.91 to 27.60]^b^ | 0.18 | |
|  |  |  |  |  |  |  |  |  |  |  |  | |
| Maternal morbidity |  |  |  |  |  |  |  |  |  |  |  | |
| Anemia intensity |  |  |  |  |  |  |  |  |  |  |  | |
| Haemoglobin, (g.dL^-1^) mean (SD) | J2 | 57 | 9.2 (1.1) | 51 | 9.1 (1.3) | 53 | 9.2 (1.1) |  |  |  |  | |
|  | J2-T0 | 38 | -1.3 (1.5) | 36 | -1.7 (1.3) | 41 | -1.3 (1.2) | -0.13  [-0.54 to 0.27] | 0.28 | -0.005  [-0.41 to 0.40] | 0.02 | |
|  |  |  |  |  |  |  |  |  |  |  |  | |
| Haemoglobin drop >4 g/dL from T0 n (%) |  | 60 | 5 (8.3) | 57 | 12 (21.0) | 58 | 10 (17.2) | 2.93  [0.95 to 9.02]^d^ |  | 2.29  [0.73 to 7.23]^d^ |  | |
|  |  |  |  |  |  |  |  |  |  |  |  | |
| Caesarean |  |  |  |  |  |  |  |  |  |  |  | |
| Duration of caesarean median [CI95%] (min) |  | 60 | 52 [44 to 71.5] | 57 | 63 [48 to 85] | 58 | 51.5 [45 to 69] | 0.09  (-0.05 to 0.23)^e^ | 0.26 | -0.02  [-0.15 to 0.12]^e^ | 0.002 | |
| General anesthesia n (%) |  | 60 | 2 (3.3) | 57 | 3 (5.3) | 58 | 3 (5.2) | NA |  | NA |  | |
|  |  |  |  |  |  |  |  |  |  |  |  | |
| Noradrenalin inotrope support n (%) |  | 60 | 8 (13.3) | 57 | 13 (22.8) | 58 | 7 (12.1) | 1.88  [0.71 to 5.01] ^d^ |  | 0.88  [0.29 to 2.63] ^d^ |  | |
|  |  |  |  |  |  |  |  |  |  |  |  | |
| Diuresis median [CI95%] (mL) | T360 | 48 | 325  [175 to 600] | 47 | 400  [(200 to 700] | 51 | 300  [160 to 600] |  |  |  |  | |
|  | T360-T0 | 46 | 200  [100 to 410] | 41 | 300  [115 to 585] | 47 | 270  [100 to 540] | 0.09  [-0.26 to 0.45]^f^ | 0.28 | 0.07  [-0.27 to 0.42] ^f^ | 0.28 | |
|  |  |  |  |  |  |  |  |  |  |  |  | |
| Transfusion |  |  |  |  |  |  |  |  |  |  |  | |
| Any Red Blood Cell transfusion n (%) | T30-D2 | 60 | 12 (20.0) | 57 | 17 (29.8) | 58 | 12 (20.7) | 1.60  [0.67 to 3.81]^d^ |  | 0.99  [0.40 to 2.47]^d^ |  | |
|  |  |  |  |  |  |  |  |  |  |  |  | |
| Red Blood Cell transfusion median [CI95%] (mL) |  | 12 | 440  [317 to 990] | 17 | 440  [221 to 495] | 12 | 440  [245 to 1100] | -4.11  [-13.32 to 5.1]^b^ | 0.35 | -3.00  [-12.97 to 6.97]^b^ | 0.25 | |
|  |  |  |  |  |  |  |  |  |  |  |  | |
|  |  |  |  |  |  |  |  |  |  |  |  | |
| Hemostatic management |  |  |  |  |  |  |  |  |  |  |  | |
| Any Procoagulant treatment n (%) |  | 60 | 16 (26.7) | 57 | 17 (29.8) | 58 | 16 (27.6) | 1.17  [0.52 to 2.63]^d^ |  | 1.05  [(0.46 to 2.37]^d^ |  | |
|  |  |  |  |  |  |  |  |  |  |  |  | |
| Fibrinogen treatment n (%) |  | 60 | 12 (20.0) | 57 | 13 (22.8) | 58 | 12 (20.7) | 1.18  [0.49 to 2.88]^d^ |  | 1.04  [0.42 to 2.47]^d^ |  | |
| Fibrinogen Dose  (g) median [95%CI] |  | 12 | 3 [3 to 4.5] | 13 | 3 [3 to 6] | 12 | 3 [3 to 4.5] | -2.52  [-10.52 to 5.47]^b^ | 0.26 | -1.37  [-9.53 to 6.78]^b^ | 0.16 | |
| Plasma treatment n (%) |  | 60 | 5 (8.3) | 57 | 3 (5.3) | 58 | 5 (8.2) |  |  |  |  | |
| Plasma Dose  (units) median [95%CI] |  | 5 | 2 [2 to 4] | 3 | 2 [4 to 8] | 5 | 3 [2 to 4] |  |  |  |  | |
|  |  |  |  |  |  |  |  |  |  |  |  | |
| Platelets treatment n (%) |  | 60 | 2 (3.3) | 57 | 2 (3.5) | 58 | 2 (3.4) | NA |  | NA |  | |
| Platelets Dose (units) |  | 2 | 1;1 | 2 | 1;2 | 2 | 1;2 | NA |  | NA |  | |
|  |  |  |  |  |  |  |  |  |  |  |  | |
| Ca+ treatment n (%) |  | 60 | 11 (18.3) | 57 | 15 (26.3) | 58 | 11 (19.0) | 1.54 [0.63 to 3.75]^d^ |  | 1.02 [0.40 to 2.60]^d^ |  | |
| Ca+ Dose  (g) median [95%CI] |  | 11 | 2 [2 to 2] | 15 | 2 [2 to 2] | 11 | 2 [2 to 2] | -0.42 [-6.46 to 5.61]^b^ | 0.05 | -3.09 [-9.58 to 3.39]^b^ | 0.60 | |
|  |  |  |  |  |  |  |  |  |  |  |  | |
| TXA treatment n (%) |  | 60 | 7 (35.0) | 57 | 5 (8.8) | 58 | 8 (13.8) | 0.73  [(0.21 to 2.46]^d^ |  | 1.21  [0.41 to 3.62]^d^ |  | |
| TXA Dose  (g) median [95%CI] |  | 7 | 1 (1 to 1) | 5 | 1 (1 to 1) | 8 | 1 (1 to 1) | NA |  | NA |  | |
|  |  |  |  |  |  |  |  |  |  |  |  | |
| Any invasive procedure n (%) |  | 60 | 16 (26.7) | 57 | 18 (31.6) | 58 | 15 (25.9) | 1.18  [0.52 to 2.6]^d^ |  | 0.91  [0.39 to 2.10]^d^ |  | |
| Intrauterine tamponadet balloon n (%) |  | 60 | 7 (11.7) | 57 | 7 (12.3) | 58 | 9 (15.5) | 1.05  [0.34 to 3.24]^d^ |  | 1.39  [0.47 to 4.04]^d^ |  | |
| Hysterectomy n (%) |  | 60 | 1 (1.7) | 57 | 1 (1.7) | 0 |  |  |  |  |  | |
|  |  |  |  |  |  |  |  |  |  |  |  | |
| Duration of Hemorrhage  min median [95%CI] |  | 60 | 29.0  [4.0 to 52.5] | 57 | 29.0  [8.0 to 49.0] | 58 | 29.0  [6.0 to 32.0] | 0.004  [-0.69 to 0.70] | 0.03 | -0.24  [-0.94 to 0.46]^b^ | 0.11 | |
|  |  |  |  |  |  |  |  |  |  |  |  | |
| Immediate postpartum events |  |  |  |  |  |  |  |  |  |  |  | |
| Hemorrhagic resurgence n(%) |  | 60 | 5 (8.3) | 57 | 3 (5.3) | 58 | 1 (1.7) | NA |  | NA |  | |
| Rescue laparoscopy n (%) |  | 60 | 2 (3.3) | 57 | 2 (3.5) | 58 | 2 (3.4) | NA |  | NA |  | |
| Oliganuria n (%) |  | 60 | 5 (8.3) | 54 | 6 (11.1) | 58 | 6 (10.3) | 1.37 [0.39 to 4.84] ^d^ |  | 1.27 [0.36 to 4.45] ^d^ |  | |
|  |  |  |  |  |  |  |  |  |  |  |  | |
| Organ failure n (%) |  | 60 | 0 | 57 | 1 (1.7) | 58 | 0 | NA |  | NA |  | |
| Intensive care unit admission n (%) |  | 60 | 7 (11.7) | 57 | 13 (22.8) | 58 | 12 (20.6) | 2.04  [0.69 to 6.06] ^d^ |  | 1.80  [0.60 to 5.43]^d^ |  | |
| Duration of intensive care unit  (hours) median [95%CI] |  | 5 | 23  [21.3 to 24.4] | 11 | 22.3  [19.1 to 29.0] | 10 | 23.7  [16.5 to 29.3] | NA |  | NA |  | |
|  |  |  |  |  |  |  |  |  |  |  |  | |
| Breastfeeding n (%) |  | 39 | 19 (48.7) | 41 | 20 (48.8) | 35 | 20 (57.1) | 1.02 (0.41 to 2.56) ^d^ |  | 1.36 (0.52 to 3.54) ^d^ |  | |
| Skin to skin n (%) |  | 43 | 31 (72.1) | 43 | 32 (74.4) | 38 | 30 (78.9) | 1.06 (0.39 to 2.90) ^d^ |  | 1.21 (0.41 to 3.55) ^d^ |  | |
|  |  |  |  |  |  |  |  |  |  |  |  | |
| Day42 postpartum data |  |  |  |  |  |  |  |  |  |  |  | |
| Hospital discharge delay (days) |  | 60 | 5 (4 to 6) | 57 | 5 (5 to 7) | 58 | 5 (4 to 6) | 0.14 (0.03 to 0.25)^e^ | 0.46 | 0.05 (-0.06 to 0.16)^e^ | 0.19 | |
| Fatigue score, mean (SD) |  | 55 | 5.6 (2.1) | 51 | 5.9 (2.4) | 54 | 5.8 (2.2) | 0.24 (-0.60 to 1.09) ^c^ | 0.11 | 0.20 (-0.63 to 1.03) ^c^ | 0.09 | |
| Experience score, mean (SD) |  | 54 | 7.1 (2.4) | 49 | 6.7 (2.2) | 54 | 6.3 (2.9) | -0.41 (-1.39 to 0.57) ^c^ | 0.16 | -0.80 (-1.75 to 0.15) ^c^ | 0.28 | |
|  |  |  |  |  |  |  |  |  |  |  |  | |
| Adverse events |  |  |  |  |  |  |  |  |  |  |  | |
| Post-Partum adverse event n (%) |  | 60 | 14 (23.3) | 57 | 26 (45.6) | 58 | 24 (41.4) | 2.76 (1.23 to 6.22)^d^ |  | 2.40 (1.06 to 5.42)^d^ |  | |
| Nausea Vomiting n (%)  Thrombosis n (%)  Increased creatinine n (%)  Renal failure n (%) |  | 60  60  60  60 | 11 (18.3)  0  2  0 | 57  57  57  57 | 10 (17.6)  0  0  0 | 58  58  58  58 | 17 (29.<8)  0  0  0 | NA  NA  NA |  | NA  NA  NA |  | |
|  |  |  |  |  |  |  |  |  |  |  |  | |
|  |  |  |  |  |  |  |  |  |  |  |  | |

Values are expressed in number (%) or median [IQR] unless otherwise indicated. Estimates [95%CI] obtained using cLDA analysis with center as random effect. NA : non applicable

^a^Estimate [95%CI] obtained using non-parametric ANCOVA adjusted on T0 values

^b^Estimate [95%CI] obtained using non-parametric ANCOVA.

^c^ Estimates [95%CI] obtained using linear mixed model with center as random effect.

^d^OR with confidence intervals obtained with logistic mixed model with center as random effect.

^e^ Estimates [95%CI] obtained using linear mixed model with center as random effect after log transformation.

^f^ Estimates [95%CI] obtained using cLDA analysis with center as random effect after a log transformation.
